# Predicting Athlete Intentions for Using Sports Complexes in the Post-Pandemic Era

**DOI:** 10.3390/ijerph20064864

**Published:** 2023-03-09

**Authors:** Tsung-Yu Chou, Peng-Yeh Lee

**Affiliations:** Department of Distribution Management, National Chin-Yi University of Technology, Taichung City 411030, Taiwan

**Keywords:** theory of planned behavior, health-promoting lifestyle, sports complex, post-pandemic era athletes

## Abstract

In recent years, the concept of health has gradually fit into people’s lives through the government’s promotion. The indoor sports complex is becoming more and more popular, offering people the opportunity to engage in physical and recreational activities regardless of weather conditions. Psychological and social abundance is the key to improving happiness, and the most important thing is to treat and care for yourself. Many fitness venues have emerged to provide athletes with a wide range of choices. However, the advent of the COVID-19 pandemic, which is caused by a virus mainly transmitted through direct contact or air droplets, has had a severe impact on indoor gym users. Therefore, based on the Theory of Planned Behavior (TPB) and Health-Promoting Lifestyle (HPL), this research investigated athletes’ behavioral intentions regarding sports halls and perceived risks as interfering variables. For data collection, we collected data samples from sports complexes athletes in Taiwan. A total of 263 responses were analyzed via SPSS 20.0 (IBM Corporation, New York, NY, USA) and AMOS 20.0 (IBM Corporation, New York, NY, USA) seis tests. The study’s results indicate that health-promoting lifestyle cognition has a positive and significant effect on behavioral intention; athletes’ attitudes, subjective norms, and perceived behavioral control significantly affects the behavioral intention of using the facilities in a sports complex. Athletes’ risk perceptions have an interference effect between HPL, attitude, subjective norm, perceived behavioral control, and behavioral intentions of using the facilities in a sports complex. Sports venue managers can refer to the results of this project to develop marketing strategies and promoting.

## 1. Introduction

With the growing domestic economy and the change of living habits and concepts, people have gradually paid more attention to sports and leisure life (Tsorbatzoudis et al. [1]). Recreational sport refers to physical activity that occurs during leisure time and focuses typically on participation, as opposed to winning material [2]. Recently, under the government’s policy of developing a public fitness program, the rate of people’s participation in sports was 80.2% in 2021, maintaining a high value of over 80% for 8 consecutive years since 2014, according to the results of the 2021 sports survey released by the Sports Administration Ministry of Education. The population participating in regular sports reached 33.9%, remaining stable at over 30.0% for eight consecutive years since 2012 [3]. However, due to the limited space for urban leisure in Taiwan, people have fewer recreational activities, leading to their lack of exercise. However, people’s health consciousness has gradually increased recently. In addition to making significant dietary adjustments, they also want to have fitness facilities for recreational activities. The Sports Complex represents a physical space for human health and fitness-centric industries that serve various societal dimensions such as cultural, economic, and social aspects at individual and national levels [4].

According to Roychowdhury [5], during the COVID-19 pandemic, the deleterious effects of physical inactivity coupled with sedentarism can concoct a dangerous recipe for a range of adverse psychophysiological health issues for individuals. This is particularly critical for the current confined circumstances, as physical inactivity and sedentarism have already been declared as global crises [6,7]. In response to this demand, sports centers began to build their indoor sports facilities to enable people to enjoy exercise without being affected by weather conditions. Many fitness complexes also emerged in communities, providing athletes with various sports and recreational activities. According to Irawan, Bastarianto, and Priyanto [8], good exercise program implementation and social distancing measures can effectively inform people’s willingness to engage in sports. Therefore, there is a need to explore athletes’ intentions for using multisport fields in the post-pandemic era.

Maglacas [9] suggested that stability and health potential are the two major dimensions of health; stability refers to a stable physical, psychological, and social balance, and health potential indicates a group or an individual’s ability to cope with environmental and psychosocial demands and stresses. Based on Richter et al. [10], the workplace can affect people’s health promotion and well-being. For people working in the sports complex, low self-perception will affect their health promotion [11].

Health promotion is not a specific disease or health-problem-prevention method, but a positive self-actualization-oriented approach that guides individuals to maintain or increase their positive attitudes toward health, self-actualization, and well-being. It represents the individual’s proactive approach to establishing new positive behaviors. Walker et al. [12] suggested that a health-promoting lifestyle is “a multifaceted, spontaneous behavior and awareness of the individual to maintain or enhance health, self-actualization, and self-fulfillment”. According to the WHO [13], health promotion enables people to increase control over and improve their health. It affects individual behaviors, environmental factors, and lifestyles. Moreover, during the COVID-19 pandemic, people are vulnerable to psychological issues, sleep disorders, fear, anxiety, depression, somatization, and obsessive-compulsive disorders [14]. This concept of a health-promoting lifestyle contributes to athletes’ attitudes towards self-health promotion, which is included in the factors that affect athletes’ behaviors towards the use of sports complexes and is one of the issues in this study.

During the consumption process, the amount of perceived risk affects the consumer’s behavior, which leads them to take certain actions that can reduce the risk [15]. According to Brown and Gentry [16], the main actions athletes take in the face of risk are buying directly and delaying or abstaining from buying to reduce the potential loss of the purchase and increase the certainty of the purchase outcome. Previous studies have discovered that athletes’ risks (corresponding to purchase intention) and perceived risks are key factors in influencing athletes’ purchase intentions, while perceived risk can significantly affect athletes’ attitudes and purchase intentions [17].

The COVID-19 pandemic emerged around the world in 2019. The virus is transmitted mainly through direct contact with viral secretions or air droplets. Up to now, tens of millions of cases have been confirmed worldwide, and the number of deaths continues to rise. This has seriously impacted the global economy, society, and the psychological well-being of people [17]. The rapid spread of COVID-19 has been attributed to the long environmental survival of the virus. It lasts in the air for 3 h, on copper surfaces for 4 h, and on cardboard surfaces for 24 h [18]. The plastic and stainless steel materials of common fitness equipment in sports centers, such as handrails, handles, and exercise equipment, are all potential vectors of the virus. Through respiratory droplets, indoor sports spaces and environments could become contaminated and cause serious impact on the facility and its clients. Therefore, the use of all gyms was suspended during the height of the pandemic. Although the pandemic is slowing down, it does not mean that people are completely at ease, as patients with no symptoms appear one after another, and the risks associated with fitness still affect indoor sports venue users. Therefore, understanding the athletes’ risk perceptions and behavioral intentions is an urgent issue for sport complex operators.

The discussion of intention and attitude began with Fishbein and Ajzen’s [19] assessment of the relationship between the two. They proposed the Theory of Reasoned Action (TRA) after considering the relevant factors affecting attitude. The authors hypothesized that individuals’ behavioral attitudes and subjective norms influence their behavioral intentions. Later, Ajzen [20] extended the (TRA) theory by suggesting that human behavior follows the principle of rational thinking, and the decision-making process goes through information gathering and comparative solution evaluation to choose the best solution. The Theory of Planned Behavior (TPB) was developed, which suggested that the intention of that behavior influences a particular behavior, and that three variables, namely, attitude, subjective norm, and perceptual behavior control, are the main factors affecting the intention of the behavior. This model is more accurate in predicting consumer behavior and has been widely used in predicting behavioral intention [17,21].

In this study, the TPB was used as a basic framework to investigate the behavior of athletes in the sports complex by combining a health-promoting lifestyle and athletes’ perceptions of risk. As described above, the objectives of this study are as follows:

(1) To investigate the influence of sports complex athletes’ behavioral attitudes, subjective norms, and perceived behavioral controls on their behavioral intentions of use.

(2) To investigate the effect of sports complex athletes’ health-promoting lifestyle awareness on their behavioral intentions of use.

(3) To investigate whether the sports complex athletes’ behavioral intentions of use are interfered with by perceived risks.

## 2. Literature Review

### 2.1. Awareness of Health-Promoting Lifestyles

Health promotion has become a hot topic in medicine, public health, and nursing. Pender et al. [22] argued that health promotion is not about disease prevention, but rather a progression that individuals or groups use to maintain and improve health, achieve self-actualization, and obtain self-fulfillment. A rather new perspective in health promotion is the capability approach, that enable people to increase control over their own lives and health [23].

It represents an individual’s active initiative to develop new behavior patterns with an approach behavior and an actualizing tendency of self-actualization toward positive growth, rather than a behavior solely directed toward disease or health problem prevention. In addition, the coaches’ efforts to promote health increase young athletes’ enjoyment, self-esteem, and health [24].

The earliest scale developed to measure HPL was the Lifestyle and Health Habits Assessment (LHHA), developed by Walker et al. [12]. They proposed that HPL is a multifaceted form of spontaneous behavior and perceptions that individuals use to enhance or maintain self-fulfillment and self-actualization. Exercise can improve physical fitness and cause energy expenditure to reduce excess fat. Therefore, correct and continuous exercise helps improve one’s body shape and keeps one physically fit [25]. The WHO [26] believes that doing sports regularly is extremely important for personal health and well-being.

In a study by Huang and Chang [27], the correlation between HPL and perceived health status was significantly positive. Moreover, exercise is the most important factor in lifestyle. It increases people’s participation in physical activities and helps them achieve better physical health through physical training [28]. Thus, it is important to exercise regularly to maintain physiological stability [29]. Heaps [30] also pointed out that exercise can improve self-image, self-satisfaction, and social adaptation while reducing anxiety, depression, and self-centeredness. Regular exercise can not only benefit people’s health but also positively affect an individual’s psychological status [31].

Regular exercise has physiological and psychological benefits; aside from promoting personal health [32], it also increases self-satisfaction and social adaptation and reduces anxiety, depression, and self-centeredness [30]. Based on past studies, regular exercise can help remove anxiety and reduce the risk of developing depression [33]. Referring to Mottola et al. [34], exercise will make people feel relaxed and comfortable as it can improve people’s body and brain temperatures.

### 2.2. Theory of Planned Behavior

The Theory of Planned Behavior (TPB) provides a theoretical framework for understanding athletes’ behavioral intentions. Ajzen [35] proposed TPB, who argued that individual behavior emerges from behavior intention. BI (behavior intention) refers to one’s intention to perform a particular behavior, i.e., the consideration that occurs before starting or preparing to engage in something [35,36]. TPB is one of the most used theoretical models for predicting people’s behaviors or behavioral intentions [37].

TPB proposes that their behavioral intention directly determines a person’s actual behavior. It is assumed that the individual’s intention to perform the behavior will be stronger if they have more positive attitudes toward a behavior, greater perception of the surrounding norm, or greater perception of control over the behavior. Therefore, the three major factors influencing athletes’ behavioral intentions are attitude, subjective norm, and perceived behavioral control, and are major predictors of behavioral intention [38,39].

Attitudes can be obtained as a psychological emotion through behavioral appraisals. BI (behavior intention) tends to be more positive if attitudes are positive [38]. The subjective norm is the sense of social pressure to perform the behavior or not, that is, the influence of other people, such as friends, relatives, or colleagues, and the feeling of social pressure on the individual to perform a particular behavior [20]. Therefore, the norm or pressure emerges when individuals consider whether others around them maintain a certain opinion about behavior before performing it, generating subjective norms [21]. Perceived behavioral control emphasizes controlling external and general factors, such as time and opportunity [40].

Therefore, based on the above, it is appropriate for this research to explore athletes’ behavioral intentions for using the facilities in a sports complex based on TPB. When an individual’s attitude towards sports promotion is more positive, their behavioral intention will be more positive, and vice versa. In terms of subjective norms, the consumer’s behavioral intentions can be predicted based on the social pressure they experience about their physical appearance. The performance of important references (e.g., family members, friends, or coworkers) would also be a source of pressure on an individual’s behavioral intentions. Perceived behavioral control reflects one’s perception of the ease of behavior [40]. The cost and time of work and the ability to access the venue’s location are also factors that influence the consumer’s behavioral intention.

### 2.3. Risk Perception

Risk is a key element in purchase behavior [41]. Perceived risk is often described as various types of possible losses or perceived uncertainty related to product selection or consumption [42]. It is a subjective perception of possible loss when seeking a desired outcome from a product or service [43]. Perceived risk has long been recognized as a key factor that influences consumer decisions and behavior [36]. While investigating how perceived risk would influence purchase intention, Cabeza-Ramírez et al. [44] discovered that perceived risk can impose a significantly negative effect on product attitude and purchase intention.

Yu et al. [45] described the perceived risk as the various perceptions of uncertainty and negative outcomes related to an athlete’s purchase or choice of a product or service. Han et al. [36] defined perceived risk as the various possible or potential losses the customer perceives in purchasing or choosing a product or service. Perceived risk in consumer behavior can be divided into multiple components depending on the nature of the loss caused by the transaction between firms and athletes [46]. Roselius [47] suggested that athletes may suffer losses in the form of time, safety, and money. Among all the concepts of perceived risk, this study defined perceived risk as the possibility of physical health problems or that people will suffer from diseases while doing exercises in sports complex during the COVID-19 pandemic.

### 2.4. Hypotheses Development

Among the diversified recreational activities, choosing the activity that suits oneself and shows one’s leisure characteristics and the ability for group cooperation can achieve the best exercise adjustment during leisure time. Exercise also mediates negative stress and generates more positive energy, restoring people’s physical and mental health, indirectly relieving stress at work, and improving interpersonal relationships and quality of life in general. Recreational activities play a neutralizing and soothing role in stress elimination [48]. Participation in sports and recreational activities is also essential for social interaction. By joining a sports community and engaging in recreational activities with others, one can build and expand their social network and enhance one’s sociability [49]. From the above literature, the cognition of HPL has a positive and significant effect on exercise and fitness behavioral intentions. Therefore, this study proposes the following hypothesis:

**H1.**  *An athlete’s health-promoting lifestyle cognition has a positive and significant effect on their behavioral intention to use the facilities in a sports complex.*

Several studies confirmed that attitudes, perceived behavioral control, and subjective norms indirectly influence intentions while directly affecting behavior [50,51]. Cabeza-Ramírez et al. [44] suggested that when an individual’s attitude is more positive, their behavioral intentions are also more positive, and vice versa. In addition, Massoud et al. [52] suggested that individuals with positive attitudes toward a particular behavior are more likely to perform the said behavior. Based on the above studies, this paper proposes the following:

**H2.**  *An athlete’s attitude significantly affects their behavioral intention of using the facilities in a sports complex.*

On the inference of subjective norms influencing athletes’ intentions, previous research focused on the extent of the impact that a significant person’s approval made on people’s intentions to perform a particular behavior [53]. Individuals’ behaviors are often based on their perceptions of others, and their intentions to accept potential behaviors are heavily influenced by those with whom they have close relationships [54]. In addition, the greater the pressure exerted by a significant person is, the greater the individual’s intention to engage in the behavior will be [55]. Based on the above studies, this paper proposes the following:

**H3.**  *An athlete’s subjective norm significantly affects their behavioral intention of using the facilities in a sports complex.*

People may not have full control over the opportunities, resources, time, knowledge, and skills available to them, but these factors influence their behavioral intentions [53], that is, the more resources and opportunities an individual believes they have and the fewer the obstacles they anticipate, the more control they will have over the behavior. Davies et al. [56] suggested that both internal factors (e.g., skills, abilities, knowledge, and proper planning) and external factors (e.g., time, opportunities, and dependence on others to cooperate) may interfere with control over expected behavior. Kautish et al. [40] also argued that behavioral control (e.g., competence) determines behavioral intentions. Thus, perceived behavioral control refers to a person’s perception of the ease of performing a particular behavior [57]. From the above, this paper proposes the following hypothesis:

**H4.**  *An athlete’s perceived behavioral control has a significant positive effect on their behavioral intention of using the facilities in a sports complex.*

Clientages perceive various types of risks due to the uncertainty associated with buying products and services. Therefore, the level of perceived risk affects their decision-making process and purchasing behavior [58]. According to Rosillo-Díaz et al. [59] perceived risk can impose a significantly negative effect on athletes’ purchase intentions. The influence of perceived risk on athletes’ purchase behavioral intentions is important in this study. Athletes perceive a higher risk in facing the outbreak of an infectious disease (e.g., COVID-19) with no clear treatment. A high level of perception leads athletes to have an intention to avoid such risks [60]. Yu et al. [61] showed that perceived risk for COVID-19 has a significant negative impact on revisiting hotels. Joo et al. [62] suggested that new infectious diseases lead to various perceived risks for athletes, which may create a psychological burden resulting in extremely passive and restrictive consumption behaviors. Therefore, the following hypotheses are proposed:

**H5.**  *An athlete’s risk perception has an interference effect between HPL and behavioral intentions of using the facilities in a sports complex.*

**H6.**  *An athlete’s risk perception has an interference effect between attitude and behavioral intentions of using the facilities in a sports complex.*

**H7.**  *An athlete’s risk perception has an interference effect between subjective norms and behavioral intentions of using the facilities in a sports complex.*

**H8.**  *An athlete’s risk perception has an interference effect between perceived behavioral control and behavioral intentions of using the facilities in a sports complex.*

## 3. Materials and Methods

### 3.1. Research Model

**Figure 1:**
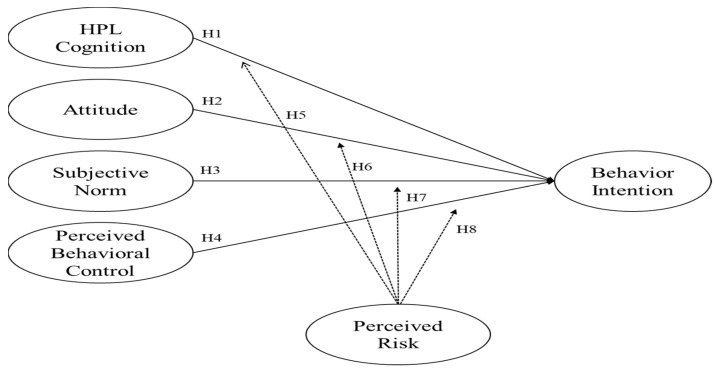
Research model.

As mentioned, this research employed the expanded TPB model to verify people’s intentions to use the sports complex. Figure 1 shows the research framework, including three TPB variables: attitude, subjective norm, perceived behavioral control, and HPL’s influence on behavior intention. Among all those variables, the perceived risk was the moderator of this study. The eight hypotheses proposed in this research are shown above.

### 3.2. Research Participants

The COVID-19 pandemic has severely hit the sport industry, and it is known that limiting physical contact is important to reduce the spread of COVID-19. In order to avoid excessive contact, users were asked to use their mobile phones to scan codes to fill out electronic forms. The random sampling method was to approach potential subjects onsite, outside of the sports complexes of three metropolitan areas in Taiwan (Taipei City, New Taipei City, and Taichung City) that were seriously affected by the epidemic from 1 September to 10 October 2022. A total of 285 questionnaires were distributed, and 263 valid questionnaires were obtained after deducting 22 invalid ones, with a recovery rate of 92%. The average time spent filling in the questionnaires was approximately 15 min, and the appropriate written consent to participant in this study was also signed in a voluntary manner by the athletes. We pointed out in Announcement No. 1010265075 of the Health Department of the Taiwan Executive Yuan that if the researchers have fully informed the subjects of the investigation of the method and purpose, the non-human test measurement method will use the questionnaire survey method.

### 3.3. Research Instruments

To measure the variables in this study, we quote effective research items from existing studies. A Likert 5-point scale measured all the items, the points on which range from 1 to 5 (strongly disagree, disagree, neutral, agree, strongly agree). The questionnaire of this research is based on the planned behavior scale compiled by Ajzen [35], the perceived risk scale by Schuett [63], and the health-promoting lifestyle (HPL) scale by Walker et al. [12]. As for the demographic analysis, gender, age, educational background, and annual income were included.

### 3.4. Data Processing and Analysis

After collecting all valid questionnaires, this study employed SPSS 20.0 to analyze the samples. Then AMOS 20.0 was used in the offending estimates, normal distribution test, confirmatory factor analysis, and structural relationship model analysis to verify the hypotheses. SEM is a statistical method used to verify assumptions and models based on the direct or indirect relationship between multiple observed and non-observed variables [64]. Moreover, it is the most appropriate way to specify measurement errors, which traditional methods cannot realize.

## 4. Results Analysis

### 4.1. Demographic Profiles

Of the 263 respondents, 46% were male and 54% were female. A total of 29 % of the participants were 41–50 years old. When asked about their educational background, 50.2% said they were university (professional), followed by postgraduate (35.7%) and high school (vocational) (14.0%). Of the participants, about 33.5% reported their annual income was between TWD 310,000–500,000, followed by TWD 510,000–700,000 (22.4%), as shown in Table 1.

### 4.2. Test of Offending Estimates

“Offending estimates” refers to a type of non-structural or measurement model. The model is improperly interpreted if the statistical coefficient exceeds the acceptable range [65]. Hence, before the overall model goodness, we would conduct the test of offending estimates in this study. Results showed that for the error variance of measured, the estimated values were between 0.02 and 0.82, all of which conformed to the standard value of 0.95 put forward by [64]. In summary, there were no offending estimates in the overall model of this study, and the overall model goodness test can be conducted later, as suggested in Table 2.

### 4.3. Normal Distribution Test

To avoid incorrect conclusions caused by the expansion of the chi-square statistic, this study referred to Kline [66] to verify standard normal distribution. In this study, no skewness of variables exceeded the absolute value of 1, and no kurtosis of variables exceeded the absolute value of 7, which means that the normal distribution has been formed in this study, as shown in Table 3.

### 4.4. Confirmatory Factor Analysis

The confirmatory factor analysis was employed in this study to verify the reliability and validity of scales [67].

#### 4.4.1. Analysis of Reliability

Reliability analytical results of this study indicate the accuracy of tools, or whether the measurement tools can be retested and have internal consistency. Cronbach’s alpha was used to check the internal consistency of questionnaire variables and the correlations between modified items and questionnaire variables. According to the results, the Cronbach’s alpha of attitude was 0.873, and its relevant modified coefficient ranged between 0.732 and 0.782; hence, all of the items were retained. The Cronbach’s alpha of subjective norm was 0.881, and its relevant modified coefficient ranged between 0.749 and 0.781; hence, all of the items remained. The Cronbach’s alpha of perceived behavioral control was 0.881, and its relevant modified coefficient was 0.761–0.780; hence, all of the items were retained. The Cronbach’s alpha of self-actualization was 0.870, and its relevant modified coefficient was between 0.592 and 0.798. Hence, all of the items remained. The Cronbach’s alpha of health responsibility was 0.815, and its relevant modified coefficient was 0.511 to 0.740; hence, all of the items were retained. The Cronbach’s alpha of sports was 0.754, and its relevant modified coefficient was within the range of 0.470 to 0.613. All of the items remained. The Cronbach’s alpha of nutrition was 0.858, and its relevant modified coefficient ranged between 0.596 and 0.804, so all the items were retained. The Cronbach’s alpha of interpersonal support was 0.815, and its relevant modified coefficient was within the range of 0.544 to 0.705. Hence, all of the items were retained. The Cronbach’s alpha of pressure treatment was 0.845, and its relevant modified coefficient ranged between 0.604 and 0.765, so all the items remained. The Cronbach’s alpha of perceived risk was 0.903, and its relevant modified coefficient was 0.795 to 0.823; hence, all of the items were retained. The Cronbach’s alpha of behavior intention was 0.735, and its relevant modified coefficient ranged between 0.496 and 0.604, so all the items were kept [67].

#### 4.4.2. Analysis of Validity

The validity analysis was based on the composite reliability (CR) of dimensions and average variance extract (AVE). Previous studies pointed out that when the value of CR is above 0.7 and the value of AVE is over 0.5, the research questionnaire has convergent validity. After conducting the convergence validity tests on the planned behavior scale, perceived risk scale, and health-promoting lifestyle scale, the test results showed that all factor loads of the dimensions were between 0.54 and 0.90, the value of CR was within the range of 0.74 to 0.88, and the value of AVE was between 0.50 and 0.72, showing good validity [67,68,69], as suggested in Table 4.

### 4.5. Analysis of Structural Relationship Model

To ensure the effectiveness of the constructed model, this study carried out the goodness test following the studies of [65,70,71,72,73,74]. Results suggested that CMIN/DF = 4.93, *p* = 0.000, GFI = 0.82, CFI = 0.82, AGFI = 0.80, RMSEA = 0.1, TLI = 0.80, and NFI = 0.80. Hence, the overall goodness of the constructed measurement model is acceptable.

#### 4.5.1. Path Analysis

The path coefficients of each dimension in this study are illustrated in Table 5. Based on the results, the standardized path coefficient of HPL on behavior intention was 0.14 (*p* = 0.026), which supported H1. The standardized path coefficient of attitude on behavior intention was 0.64 (*p* < 0.000), which verified H2. The standardized path coefficient of subjective norm on behavior intention was 0.29 (*p* = 0.027), which proved H3. The standardized path coefficient of perceived behavioral control on behavior intention was 0.35 (*p* = 0.009), which proved H4.

#### 4.5.2. Analysis of Moderating Effects of Perceived Risk

As for the moderating effects of perceived risk adjustment, compared with other research methods such as ANOVA or regression analysis, SEM can measure the influence of errors and estimate the main moderating effects with higher explanatory power [75]. Although this study only focused on the moderating effect of potential continuous variables, if the moderator is an explicit category variable, the data can be divided into sub-groups and processed by Structural Equation Modeling [76]. The analysis of moderating effects of perceived risk was suggested in Table 5. The standardized path coefficient of the effect of perceived risk between HPL and behavior intention was −0.78 (*p* = 0.009), proving H5. The standardized path coefficient of the effect of perceived risk between attitude and behavior intention was 0.20 (*p* = 0.026), which proved H6. The standardized path coefficient of the effect of perceived risk between subjective norm and behavior intention was 0.34 (*p* = 0.022), which proved H7. The standardized path coefficient of the effect of perceived risk between perceived behavioral control and behavior intention was 0.32 (*p* = 0.019), which proved H8.

## 5. Discussion and Implications

The widespread of COVID-19 highlighted the importance of human behaviors in controlling disease transmission. At the start of the COVID-19 pandemic, people were not vaccinated. Nondrug preventive measures, such as wearing masks, washing hands, and keeping social distance, are important and cost-effective ways to control the pandemic. During the COVID-19 pandemic, the government of Taiwan closed indoor sports halls, bringing considerable difficulties to the practitioners. Even though the situation has improved during the post-pandemic era, the number of returned athletes is not as much as before.

Hammerschmidt, Durst, Kraus, and Puumalainen’s [77] research shows that the biggest problem for clubs during COVID-19 is liquidity, and the crisis brought by COVID-19 will quickly threaten their operations [78]. Therefore, it is urgent to discuss clubs and athletes. This study was employed to strengthen the concept of behavior role exploration previously conducted by [35,79], enabling sports complex and post-pandemic era athletes to develop and build favorable interactive relationships.

In this research, a complete TPB model was developed to explain the key structure for athletes in deciding the intention of using sports complexes during COVID-19, and the model was further improved to be more complete through HPL and perceived risk. Based on the results, athletes’ attitudes have the greatest impact on behavior intention, and the hypotheses relating to athletes’ intentions of using sports complexes have been verified. Most importantly, referring to this study, while an athlete’s attitude, subjective norm, perceived behavioral control, and HPL affect their willingness to use sports complexes, perceived risk can moderate an athlete’s intention to use sports complexes.

### 5.1. Theoretical Implications

First, this study effectively added HPL into the TPB model to explore the athletes’ behavior intentions of using sports complexes. Past studies mainly investigated the direct effects of attitude, subjective norm, and perceived behavioral control on behavior intention. As the public usually has expectations and requirements for their health before using the sports complex, it is an important innovation for this study to add athletes’ perceptions of HPL on behavior intentions.

Secondly, according to the results, perceived risk is crucial to an athlete’s behavior intention. Moreover, perceived risk significantly modifies health-promoting lifestyle cognition, attitude, subjective norm, perceived behavioral control, and behavior intention. Among them, the most influential is the moderating effect of health-promoting lifestyle cognition on behavior intention. We discovered that athletes’ use of sports complexes can be moderated by COVID-19. The results showed that COVID-19 has greatly affected and changed the lifestyle of athletes and has caused a considerable impact on the use of sports complexes. Hence, this study can also be a reference for operators of sports complexes.

Finally, this study acted as an important reference to identify factors affecting athletes’ intentions to use sports complexes. Based on the results, attitude can significantly impact athletes’ visits to the sports complex. Moreover, past studies have revealed the influence of attitude on behavior intention [41], which indicated that when the feelings of athletes about using sports venues are more valuable, interesting, or beneficial, they will be able to stimulate a focus variable to use sports complexes. As for health-promoting lifestyle cognition, its impact on behavior intention is not the biggest, but with the moderator of risk cognition, its impact can be negative, as indicated in previous research reports [36,80]. Hence, it can be concluded that, due to COVID-19, the original positive impacts of athletes’ self-actualization, health responsibility, sports activities, interpersonal support, and pressure on athletes’ intentions of using sports complexes can be transformed into negative results. This noteworthy finding indicated that, although these athletes have the correct awareness of health-promoting lifestyle and are ready to enter sports complexes for exercise, they are reluctant to do so as COVID-19 weakens their universal intention [48,49].

### 5.2. Practical Implications

The results affected the management of sports complexes. First, it will be valuable to improve athletes’ attitudes about sports complexes or to promote sports education. In addition, improving athletes’ sports values and attitudes can be an important factor in triggering behavior intention. Even so, managers of sports complexes or centers should be quite cautious to avoid leaving bad impressions by exaggerating their words. The challenges faced by those managers is to provide athletes with an information balance during the marketing, and allow athletes to experience activities honestly and transparently.

Moreover, this study discovered that perceived risk can affect users’ behavior intentions, which can be an important reminder for complex sports managers. In other words, athletes’ attitudes, subjective norms, perceived behavioral control, and health-promoting lifestyle cognition positively affects the sports complex’s behavioral intentions. However, these variables will adjust athletes’ behavior intentions based on their perceived risks caused by virus infection. For example, according to Zhang et al. [81], through high perceived risks, even high control levels, high subjective norms, or original behaviors can be altered due to fear. Furthermore, since our study has revealed the important role of perceived risk, the government and enterprises should act to reduce athletes’ perceived risks in the use of sports complexes during the post-pandemic era. The government should advocate the right anti-pandemic measures to lower the risk of exercise for athletes, while practitioners should perform various cleaning and disinfection policies to improve the athletes’ sense of safety. More specifically, the government can use mass media to spread related norms that should be followed during the exercise process, making athletes feel certain about not acquiring an obtaining infection by using correct prevention concepts. For enterprises, they should strictly enforce government regulations and continue to provide adequate guarantees for the safety of sports complexes and facilities to attract more customers.

Thirdly, according to the results, managers can formulate effective marketing strategies based on athletes’ health-promoting lifestyle cognition to improve athletes’ behavior intentions. For instance, Nimri et al. [82] emphasized the key role of environmental protection in affecting individuals’ decisions to choose green hotels. Therefore, marketing personnel of sports centers should carry out advertising activities to advocate the importance of a health-promoting lifestyle, which will make athletes believe that they should exercise, considering their demands for a healthy lifestyle.

### 5.3. Limitations and Future Research

As this study only targeted the athletes of Taiwanese sports complexes, future studies can make a cross-comparison to focus on cross-cultural athletes. Additionally, each operating model of a sports complex is special, and there are various kinds of sports complexes in Taiwan. This study only touched on the behavior intention of athletes towards sports complexes, but how the athletes react when located in different operating models can also be worthy of future discussion.

Moreover, under the impact of COVID-19, whether the moods of athletes and operators can adapt to the changes, and whether athletes’ requirements for operators can be changed into the consumption mode before the COVID-19 when COVID-19 is tamed, all deserve investigation. It is suggested that the follow-up research can conduct multiple comparisons on how different sports complexes can affect athletes’ behavior intentions.

Additionally, in the future, studies can evaluate whether the results of this research can still be effective if the external environment changes, which can provide more in-depth business insights into the management of sports complexes. Since athletes’ behavioral intentions for sports complexes were revealed, future research can re-examine what products or services are essential for athletes and the price rationality they agree with to provide athletes with acceptable leisure and entertainment products and sports services.

## 6. Conclusions

In this research, the following research conclusions are obtained Theory of Planned Behavior (TPB) and Health-Promoting Lifestyle (HPL), investigating athletes’ behavioral intentions regarding sports halls and perceived risks as interfering variables. The Health-Promoting Lifestyle cognition, attitudes, subjective norms, and perceived behavioral control has a positive and significant effect on behavioral intention; The risk perceptions have an interference effect between HPL, attitude, subjective norm, perceived behavioral control, and behavioral intentions.

## Figures and Tables

**Table 1:** Analysis of sample characteristics.

Variable	Category	Count
Gender	Male	121
	Female	142
Age	20 years and below	43
	21–30 years	46
	31–40 years	45
	41–50 years	77
Education level	51–60 years	44
	61 and above	8
	High school	37
	University	132
	Postgraduate schools	94
Yearly family income	TWD 300,000 below	43
	TWD 310,000–500,000	88
	TWD 510,000–700,000	59
	TWD 710,000–900,000	28
	TWD 900,000 above	45

Note: the hourly minimum wage TWD 168 is of 31 December 2022.

**Table 2:** The test of offending estimates.

Construct	Variable	Standardized Regression Coefficient	Error Variance
Attitude	A1	0.80	0.11
	A2	0.84	0.10
	A3	0.87	0.10
Subjective norm	SN1	0.86	0.73
	SN2	0.86	0.73
	SN3	0.81	0.82
Perceived behavioral control	PBC1	0.84	0.08
	PBC2	0.83	0.07
	PBC3	0.86	0.08
Self-actualization	SA1	0.64	0.04
	SA2	0.90	0.07
	SA3	0.84	0.09
	SA4	0.79	0.07
Health responsibility	HR1	0.74	0.05
	HR2	0.74	0.06
	HR3	0.84	0.03
	HR4	0.61	0.02
Exercise	EX1	0.80	0.02
	EX2	0.54	0.02
	EX3	0.70	0.04
	EX4	0.70	0.05
Nutrition	NU1	0.67	0.03
	NU2	0.73	0.03
	NU3	0.88	0.05
	NU4	0.87	0.06
Interpersonal support	IS1	0.82	0.06
	IS2	0.75	0.04
	IS3	0.78	0.05
	IS4	0.57	0.05
Stress management	SM1	0.67	0.02
	SM2	0.70	0.03
	SM3	0.86	0.02
	SM4	0.85	0.03
Perceived risk	PR1	0.82	0.40
	PR2	0.87	0.40
	PR3	0.85	0.34
Behavior intention	BI1	0.81	0.23
	BI2	0.60	0.20
	BI3	0.66	0.19

**Table 3:** The normal distribution.

Variable	Min	Max	Skew	c.r.	Kurtosis	c.r.
A1	1	5	−0.247	−1.637	−1.571	−5.199
A2	1	5	−0.432	−2.863	−1.368	−4.529
A3	1	5	−0.498	−3.297	−1.293	−4.28
SN1	1	5	−0.476	−3.154	−1.067	−3.531
SN2	1	5	−0.423	−2.802	−1.051	−3.48
SN3	1	5	−0.354	−2.344	−1.218	−4.033
PBC1	1	5	−0.343	−2.271	−1.148	−3.801
PBC2	1	5	−0.45	−2.982	−1.015	−3.36
PBC3	1	5	−0.559	−3.699	−1.012	−3.351
SA1	1	5	−0.572	−3.784	0.367	1.215
SA2	1	5	−0.51	−3.374	0.364	1.204
SA3	1	5	−0.505	−3.341	0.382	1.264
SA4	1	5	−0.511	−3.384	0.114	0.378
HR1	1	5	−0.363	−2.401	0.276	0.913
HR2	1	5	−0.229	−1.513	−0.25	−0.829
HR3	1	5	−0.574	−3.798	0.67	2.217
HR4	2	5	−0.255	−1.686	−0.496	−1.643
EX1	1	5	−0.327	−2.165	−0.424	−1.405
EX2	1	5	−0.242	−1.601	−0.091	−0.301
EX3	1	5	−0.11	−0.731	−0.409	−1.355
EX4	1	5	−0.791	−5.238	0.764	2.531
NU1	1	5	−0.588	−3.895	0.487	1.612
NU2	1	5	−0.304	−2.014	−0.339	−1.123
NU3	1	5	−0.59	−3.903	0.786	2.601
NU4	1	5	−0.489	−3.235	0.124	0.411
IS1	2	5	−0.359	−2.375	−0.192	−0.634
IS2	1	5	−0.669	−4.426	0.885	2.929
IS3	2	5	−0.453	−2.996	−0.195	−0.645
IS4	1	5	−0.429	−2.84	0.008	0.025
SM1	1	5	−0.499	−3.302	0.675	2.235
SM2	1	5	−0.61	−4.037	0.566	1.872
SM3	2	5	−0.528	−3.496	0.101	0.334
SM4	2	5	−0.575	−3.808	−0.005	−0.017
PR1	1	5	0.897	5.939	0.795	2.633
PR2	1	5	1.052	6.967	1.11	3.676
PR3	1	5	0.966	6.394	1.096	3.629
BI1	1	5	−0.273	−1.807	−1.587	−5.254
BI2	1	5	−0.468	−3.099	−1.509	−4.994
BI3	1	5	−0.391	−2.588	−1.484	−4.912

**Table 4:** The verification analysis of facets.

Construct	Pointer	Standardized Factor Loading	Unstandardized Standardized Factor Loading	S.E.	C.R. (t-Value)	*p*	SMC	C.R.	AVE
Attitude	A1	0.80	1				0.640	0.88	0.71
	A2	0.84	0.994	0.07	14.11	***	0.706		
	A3	0.87	1.042	0.072	14.394	***	0.757		
Subjective norm	SN1	0.86	1				0.740	0.88	0.71
	SN2	0.86	1.008	0.065	15.614	***	0.740		
	SN3	0.81	0.986	0.066	14.884	***	0.656		
Perceived behavioral control	PBC1	0.84	1				0.706	0.88	0.71
	PBC2	0.83	0.949	0.063	14.979	***	0.689		
	PBC3	0.86	1.02	0.066	15.353	***	0.740		
Self-actualization	SA1	0.64	1				0.410	0.87	0.64
	SA2	0.90	1.481	0.128	11.611	***	0.810		
	SA3	0.84	1.392	0.127	10.998	***	0.706		
	SA4	0.79	1.202	0.112	10.711	***	0.624		
Health responsibility	HR1	0.74	1				0.548	0.83	0.54
	HR2	0.74	1.093	0.093	11.709	***	0.548		
	HR3	0.84	1.056	0.08	13.164	***	0.706		
	HR4	0.61	0.658	0.072	9.154	***	0.372		
Exercise	EX1	0.80	1				0.563	0.78	0.50
	EX2	0.54	0.696	0.084	8.253	***	0.292		
	EX3	0.70	0.964	0.09	10.711	***	0.476		
	EX4	0.70	0.781	0.077	10.156	***	0.436		
Nutrition	NU1	0.67	1				0.449	0.87	0.63
	NU2	0.73	1.159	0.112	10.333	***	0.533		
	NU3	0.88	1.194	0.097	12.314	***	0.774		
	NU4	0.87	1.286	0.106	12.136	***	0.757		
Interpersonal support	IS1	0.82	1				0.672	0.82	0.54
	IS2	0.75	0.989	0.08	12.331	***	0.563		
	IS3	0.78	0.897	0.07	12.809	***	0.608		
	IS4	0.57	0.844	0.096	8.759	***	0.325		
Stress management	SM1	0.67	1				0.449	0.86	0.60
	SM2	0.70	0.929	0.094	9.902	***	0.490		
	SM3	0.86	1.137	0.096	11.844	***	0.740		
	SM4	0.85	1.202	0.104	11.6	***	0.723		
Perceived risk	PR1	0.82	1				0.672	0.88	0.72
	PR2	0.87	1.081	0.072	14.989	***	0.757		
	PR3	0.85	0.97	0.066	14.779	***	0.723		
Behavior intention	BI1	0.81	1				0.656	0.74	0.50
	BI2	0.60	0.754	0.109	6.91	***	0.360		
	BI3	0.66	0.815	0.115	7.074	***	0.436		

Note: *** *p* < 0.001.

**Table 5:** Results of hypotheses testing.

Hypotheses	Path	Standardized Path Coefficient	Test Results
H1	HPL → BI	0.64 ***	Supported
H2	At → BI	0.29 ***	Supported
H3	SN → BI	0.35 ***	Supported
H4	PC → BI	0.14 ***	Supported
H5	HPL × PR → BI	−0.78 ***	Supported
H6	At × PR → BI	0.20 ***	Supported
H7	SN × PR → BI	0.34 ***	Supported
H8	PBC × PR → BI	0.32 ***	Supported

Note: *** *p* < 0.001.

## Data Availability

Data is available on request from corresponding author.
